# Association of Plasma Neurofilament Light Chain With Glycaemic Control and Insulin Resistance in Middle-Aged Adults

**DOI:** 10.3389/fendo.2022.915449

**Published:** 2022-06-20

**Authors:** Rohith N. Thota, Pratishtha Chatterjee, Steve Pedrini, Eugene Hone, Jessica J. A. Ferguson, Manohar L. Garg, Ralph N. Martins

**Affiliations:** ^1^ Macquarie Medical School, Macquarie University, North Ryde, NSW, Australia; ^2^ School of Biomedical Sciences and Pharmacy, University of Newcastle, Callaghan, NSW, Australia; ^3^ School of Medical and Health Sciences, Edith Cowan University, Joondalup, WA, Australia; ^4^ Australian Alzheimer’s Research Foundation, Nedlands, WA, Australia; ^5^ School of Psychiatry and Clinical Neurosciences, University of Western Australia, Crawley, WA, Australia; ^6^ The KaRa Institute of Neurological Disease, Macquarie Park, NSW, Australia

**Keywords:** biomarkers, cross-sectional study, insulin resistance, neurodegeneration, neurofilament light chain, type 2 diabetes

## Abstract

**Aims:**

This study aimed to determine the association of plasma neurofilament light (NfL), a marker of neurodegeneration, with diabetes status and glycaemic parameters in people with normal glycaemia (NG), pre-diabetes (PD) and type 2 diabetes (T2D).

**Methods:**

Clinical and descriptive data for the diagnostic groups, NG (n=30), PD (n=48) and T2D (n=29), aged between 40 and 75 years were included in this cross-sectional analysis. Plasma NfL levels were analyzed using the ultra-sensitive single-molecule array (Simoa) platform.

**Results:**

A positive correlation was evident between plasma NfL and fasting glucose (r = 0.2824; p = 0.0032). Plasma NfL levels were not correlated with fasting insulin and insulin resistance. Plasma Nfl levels were significantly different across the diabetes groups (T2D >PD >NG, p=0.0046). *Post-hoc* analysis indicated significantly higher plasma NfL levels in the T2D [12.4 (5.21) pg/mL] group than in the PD [10.2 (4.13) pg/mL] and NG [8.37 (5.65) pg/mL] groups. The relationship between diabetes status and NfL remained significant after adjusting for age, sex, BMI, HOMA-IR and physical activity (adjusted r^2^ = 0.271, p = 0.035).

**Conclusions:**

These results show biomarker evidence of neurodegeneration in adults at risk or with T2D. Larger sample size and longitudinal analysis are required to better understand the application of NfL in people with risk and overt T2D.

## Introduction

Diabetes mellitus (DM) is associated with complications involving the retina, kidneys, and peripheral nerve (1). Exceptional control of diabetes-related hyperglycaemia may slow the progression or prevent the development of diabetic complications altogether, however, the reversal of complications does not occur. Among these complications, DM selectively targets the peripheral nervous system in a widespread or diffused fashion leading to polyneuropathy or selectively causing focal neuropathies (diabetic neuropathy) (2). Existing evidence suggests that this neuropathy can develop in both pre-diabetes and an overt DM state (3). The prevailing view of the underlying pathophysiology is axonal degeneration and progressive loss of nerve fibres (4). Procedures including needle examination and skin punch biopsies are often required for the diagnosis of neuropathy when small fiber injury is the sole pathology (5) and can often be painful and inconvenient to the patient. It is essential for research and clinical practice to have a feasible test that can reliably detect and monitor such damage.

Neuro-axonal damage is a consequence of many neurological diseases such as multiple sclerosis, stroke, dementias, including Alzheimer’s disease (AD) and contributes to cognitive deficits and physical disability (5). Evidence from epidemiological, clinical and animal studies has established strong links between type 2 diabetes (T2D) and neurodegeneration (6–8). The key mechanisms that underpin these are evolving rapidly (9). Still, current evidence links to the involvement of insulin signalling dysfunction (10, 11), inflammatory and oxidative stress (12), pathways that occur early in the neurodegenerative process (13). Recently, neurofilament (Nf) protein, a neurodegeneration marker, has gained increasing attention in this space. Nf is a crucial axonal cytoskeletal component comprising neurofilament light chain (NfL), neurofilament medium chain (NfM), and neurofilament heavy chain (NfH) (14). The disruption of Nf in neuronal damage occurring within neurodegenerative conditions results in the release of Nf into the cerebrospinal fluid (CSF), consequently giving rise to elevated NfL concentrations in the CSF and blood, as seen in neurodegenerative diseases (15–17). Until recently, studies were limited to CSF NfL, because detection systems were not sensitive enough to quantitate the physiologically lower levels of NfL in the blood (18). This has changed with the introduction of the single-molecule array (Simoa) technology (19), which now provides the analytical basis for highly sensitive quantitation of the NfL subunit in the blood (20). Several studies have demonstrated high correlation between CSF and plasma NfL levels, and this has given reason to study NfL in a wide range of neurological disorders (21). Higher plasma NfL levels have been associated with compromised cognition and hippocampal atrophy in both cross-sectional and longitudinal human studies (22). Plasma NfL levels were also inversely associated with brain glucose metabolism, longitudinally in the highly characterized Alzheimer’s Disease Neuroimaging Initiative (ADNI cohort) (23). A previous report indicated a positive correlation between NfL mRNA levels and Douleur Neuropathique 4 questionnaire score in pre-diabetic patients, indicating an early detection marker of pre-diabetic peripheral neuropathy (24). Therefore, plasma levels of NfL may be an attractive indicator of neurodegeneration in people with different glycaemic status. The current study aimed to investigate the association of plasma NfL levels with glycaemic control and diabetes status in a cross-sectional study designed to provide initial evidence of plasma NfL levels as a possible marker for early detection of diabetic neuropathy.

## Methodology

### Participants

Participants with normal glycaemic control (NG), pre-diabetes (PD), and T2D were recruited from the Newcastle and Hunter region (New South Wales, Australia) by radio announcements, newspaper articles and advertisements placed around the local community. Our investigation was based on data and baseline blood samples obtained from the ‘curcumin and omega-3 fatty acids for the prevention of T2D’ (COP-D trial, ACTRN12615000559516) for individuals with pre-diabetes, ‘curcumin and omega-3 fatty acids for the management of dyslipidaemia in T2D’ (CALFOR-CVD trial, ACTRN12616001483448) for individuals with T2D, and ‘plant sterols and curcumin for the prevention of cardiovascular disease’ (PAC-CVD trial, ACTRN12615000956505) for individuals with normal plasma glucose levels. The detailed methodology of these studies have been previously published (25–27). The mean duration of diabetes was 6.0 ± 0.9 years in people with T2D, and there were no diabetic complications reported in these patients at the time of recruitment. Seventy four percent of the total participants in T2D group were taking oral anti-hyperglycaemic medications along with 67% on anti-hypertensive medications, 55% on cholesterol lowering medications and 11% on over-the-counter supplements. From all recruited subjects, participants aged between 40-75 and those recruited between 2016-2017 were included in the final analysis for the current study (n=107). The study protocols for all three clinical trials were approved by the Human Research Ethics Committee of the University of Newcastle, Callaghan, Australia, and written informed consent was obtained from all participants.

### Blood Samples

Fasting blood samples (10 h) were collected into EDTA vacutainers *via* venipuncture by a trained phlebotomist and plasma fractions were obtained by centrifuging (Heraeus Biofuge Stratos) for 10 min at 3000 ×g at 4°C. Plasma samples were aliquoted and stored at −80°C until further analysis. Fasting plasma glucose was measured on a VP auto analyzer using Pathology North Services standardized reagents. Insulin was measured by chemiluminescent microparticle immunoassay (CMIA), Abbott Architect method, by Pathology North service.

### Insulin Resistance and Body Weight Measurements

Insulin resistance was estimated using the homeostatic model assessment (HOMA-IR). This is based on the formula fasting glucose (mmol/l) X fasting insulin (µIU/L)/22.5. The height of the participants was measured using a wall-mounted stadiometer with a movable headpiece. Weight and BMI were calculated using bioelectrical impedance (BIA) (InBody230, Biospace Co.). Measurements were collected in the fasting state whilst standing. Participants refrained from vigorous physical activity and alcohol consumption 24 h before data collection. A physical activity questionnaire (International Physical Activity Questionnaire; IPAQ Long Form Last 7 Days Self-Administered Format, October 2002) was used to collect physical activity data.

### NfL Measurements

All plasma samples were analyzed by ultra-sensitive single-molecule array (Simoa) assay at Edith Cowan University, Western Australia, Australia, following manufacturer’s instructions (28). Calibrators, internal controls and samples were run in duplicates. Internal controls and samples were diluted fourfold.

### Statistical Analysis

Statistical analysis was performed using Stata/IC 14.2 (StataCorp LP, Texas, USA). Shapiro-Wilk test was employed to test the assumption for normality. Normally distributed variables are reported as mean ± SEM and non-normally distributed variables as median (Interquartile range, IQR). Pearson correlation for normally distributed data and Spearman correlation for non-normally distributed data were employed for correlation analyses. For comparison across groups, Analysis of variance (ANOVA) was used for normally distributed data and the Kruskal Wallis test for non-normally distributed data. For significant effects, Tukey’s HSD or Dunn’s test was used to perform *post hoc* comparisons to test the difference in the dependable variables between NG, PD, and T2D groups. Log transformation was applied to obtain the normality for NfL levels and other non-parametric variables. Multiple regression models were established to determine independent determinants of NfL levels. Unadjusted and adjusted (for age, sex, BMI, HOMA-IR and physical activity) regression analyses were carried out for NfL and diabetes status (NG, PD, and T2D). A two-sided p-value (p)< 0.05 was considered to be statistically significant.

## Results

Clinical and descriptive data for the diagnostic groups, NG (n=30), PD (n=48) and T2D (n=29) are presented in **Table 1**. The plasma glucose levels were significantly higher in the PD [5.6(1) mmol/L] and T2D [7.8(2.5) mmol/L] group than in the NG group [5(0.6) mmol/L]. Similarly, fasting insulin levels were significantly higher in people with PD [10.1(7.6) mIU/L] compared to NG. However, there were no differences observed in the HOMA-IR levels between the PD and NG groups **(** **Table 1** **)**. The T2D group [3.8(3.2)] had significantly higher insulin resistance than the PD and NG group. Participants in the NG group [52(14) years] were significantly younger than those in T2D group [67(14)], but there was no statistically significant difference between the age of participants in the NG and PD groups. Compared to NG, BMI was significantly higher in PD and T2D **(** **Table 1** **)**. Physical activity levels were significantly lower in the PD and T2D groups than in the NG group **(** **Table 1** **)**.

**Table 1:** Participant characteristics.

Parameters	Normoglycaemia (n=30)	Pre-diabetes (n=48)	Type 2 diabetes (n=29)
**Age (years)**	52 (14)^a^	59 (15)	67 (14)^a^
**Sex (m/f)**	14/16	20/28	19/10
**Body mass index (kg/m^2^)**	27.6 ± 0.9^b,c^	31.4 ± 0.7^b^	31.5 ± 1.1^c^
**Waist circumference (cm)**	93 ± 2.2^d,e^	104.2 ± 1.7^d^	112 ± 2.8^e^
**Skeletal muscle mass (kg)**	30.5 ± 1.5^f^	54.7 ± 1.6	34.4 ± 1.4^f^
**Fat mass** **(kg)**	25.4 ± 2.0^g^	33.4 ± 1.7^g^	32.5 ± 2.5
**Fasting plasma glucose (mmol/L)**	5 (0.6)^h,i^	5.6 (1)^h^	7.8 (2.5)^i^
**Fasting plasma insulin (µIU/L)**	6.8 (3.8)^j,k^	10.1 (7.6)^j^	10.8 (8.2)^k^
**HOMA-IR**	1.4 (1.2)^l^	1.4 (1.2)^m^	3.8 (3.2)^l,m^
**Total cholesterol**	6.2 ± 0.2^n,o^	5.7 ± 0.2^n^	4.7 ± 0.2°
**Total : HDL-Cholesterol**	4.7 ± 0.2	4.4 ± 0.2	4.3 ± 0.2
**Triglycerides**	1.75 (0.7)^p^	1.5 (0.5)	2.4 (0.8)^p^
**CRP**	3.1 (2.3)	3.8 (2.7)	4.1 (3)
**Physical activity (MET-minutes/week)**	3402 (5907)^k^	2944 (4087)^l^	1710 (2016)^k,l^

Data is presented as mean ± SEM or median (Interquartile range). HOMA-IR, homeostatic model assessment for insulin resistance; Physical activity is measured using International Physical Activity Questionnaire – Long Form, version 2002. Significance is set at P < 0.05. Mean or medians with the same small letter represent significant (p < 0.05) differences.

### Plasma NfL Levels in NG, PD and T2D Groups

**Figure 1:**
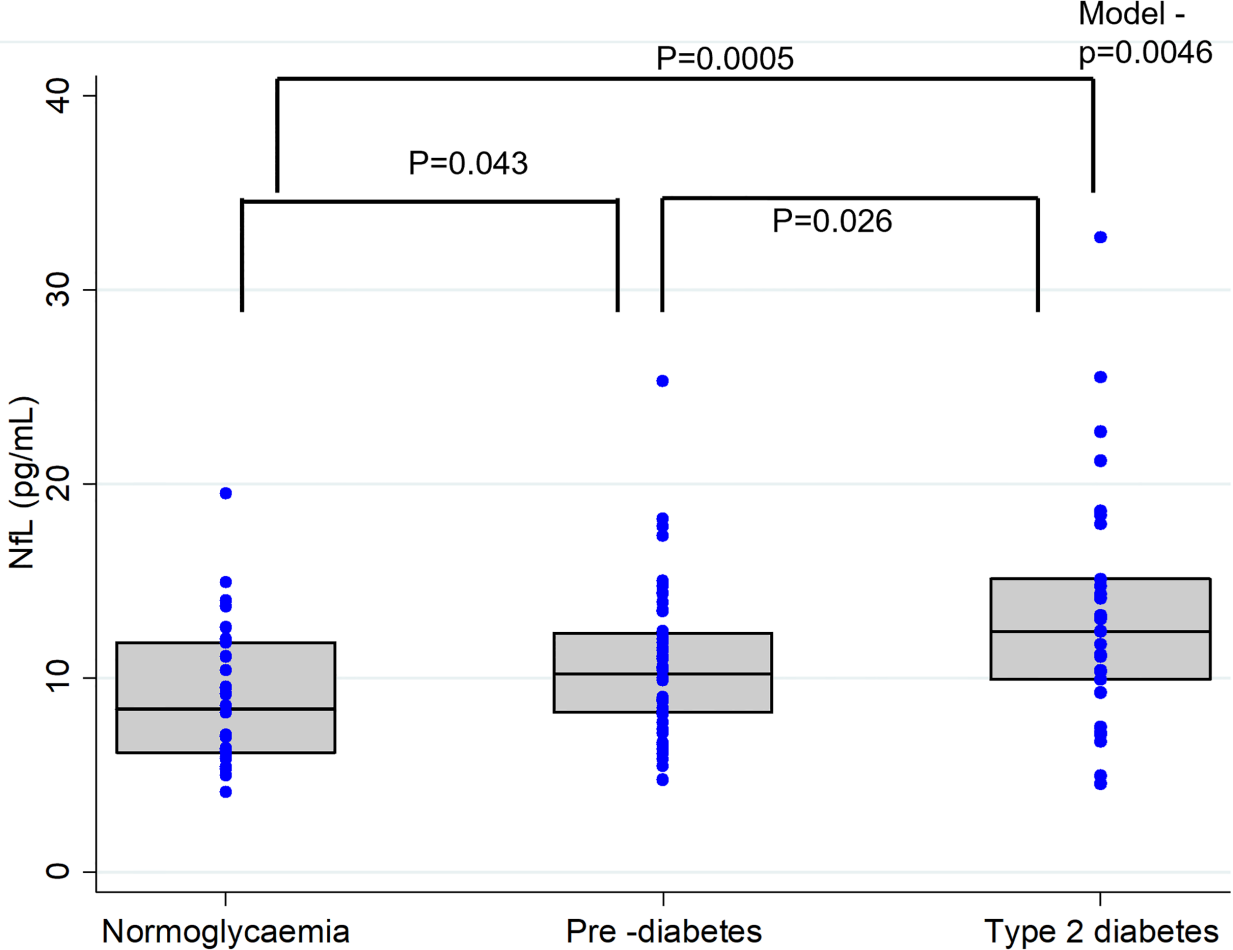
Plasma Neurofilament light (NFL) concentrations in individuals with normal glucose levels, pre-diabetes and type 2 diabetes. In the box-whisker plots central bar indicate the median NFL concentration and upper and lower boundaries. Blue dots represent the NFL data points. P-values representing differences in plasma NfL levels between the groups were obtained from Post-hoc analysis (Dunn's test).

Plasma NfL levels were significantly different across the diabetes groups (T2D > PD > NG, *P*=0.0046). *Post-hoc* analysis indicated significantly higher plasma NfL levels in the T2D [12.4 (5.21) pg/mL] group than the PD [10.2 (4.13) pg/mL] and the NG [8.37 (5.65) pg/mL] groups **(** **Figure 1** **)**. Similarly, compared to the NG group, plasma NfL levels were significantly elevated in the PD group **(** **Figure 1** **)**.

### Correlation of Plasma NfL Levels and Study Outcomes

**Figure 2:**
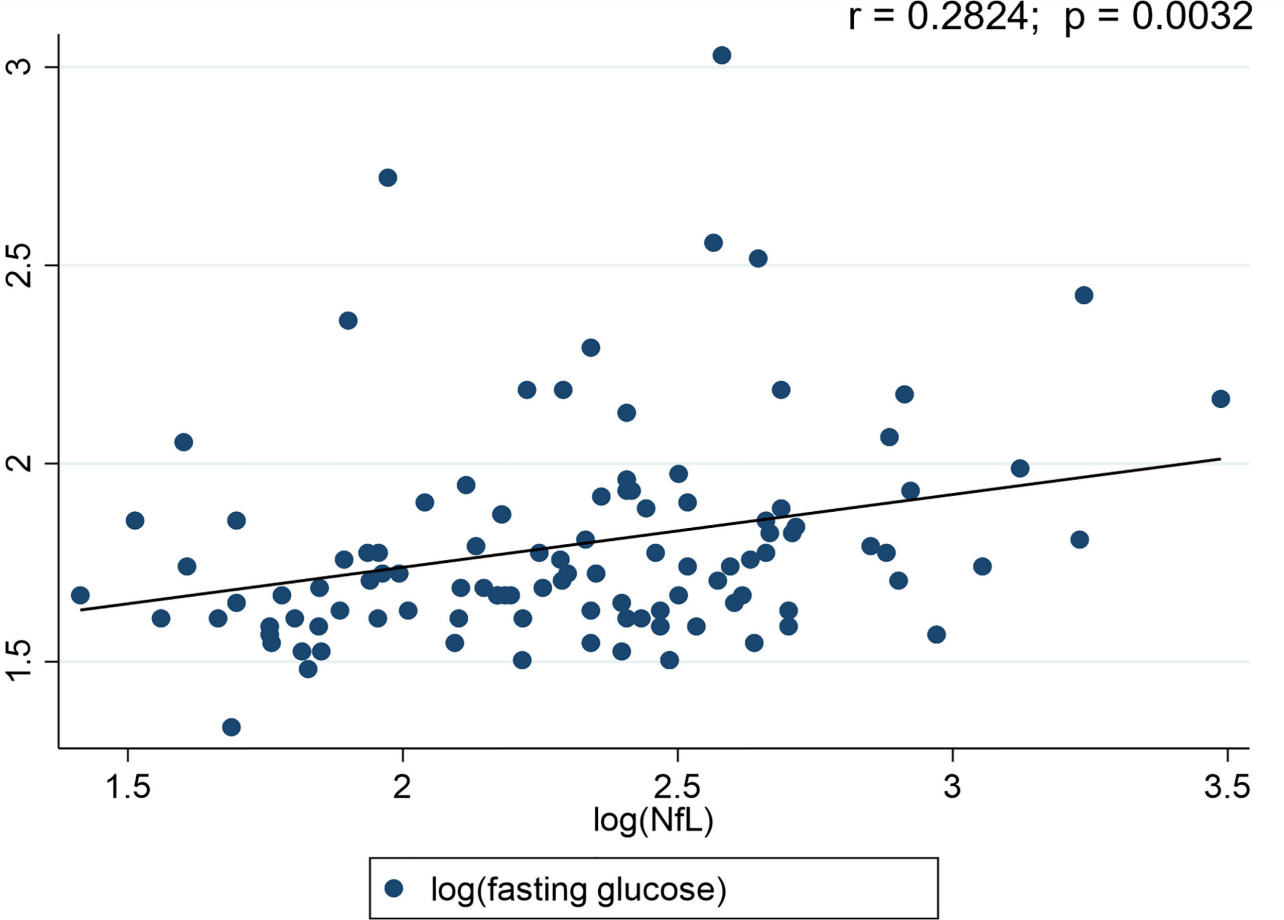
Scatter plots for Neurofilament light chain (NfL) and diabetes status. r-value represents the correlation between the blood glucose levels and NfL.

A significant positive correlation (r = 0.5038; p < 0.0001) was detected between the participants’ age and their plasma NfL levels. Plasma NfL correlated (r = 0.2824; p = 0.0032) with plasma fasting glucose levels **(** **Figure 2** **)**. However, no significant (p>0.05) associations were observed for fasting plasma insulin (r = -0.0356), HOMA-IR (r = 0.0665), BMI (r = -0.0504) and physical activity (r = 0.1511) with plasma NfL levels.

### Linear Regression Models for Predictors of Plasma NfL

Unadjusted regression analysis indicated that participants’ diabetes status (i.e., NG, PD, T2D) was a significant predictor of plasma NfL levels **(** **Table 2** **)**. However, categorical analysis showed that only T2D diagnosis (β-coefficient= 0.366, p= 0.001), over PD and NG, predicted plasma NfL levels. T2D diagnosis accounts for 36% increase in the outcome variable, plasma NfL levels. Participants’ age and plasma glucose levels also significantly predicted plasma NfL levels **(** **Table 2** **)**. Significance of diabetes status (adjusted r^2 =^ 0.271, p = 0.035) remained after adjusting for age, sex, BMI, HOMA-IR and physical activity levels **(** **Table 2** **)**. When adjusted for CRP (as a marker of systemic inflammation), both pre-diabetes and diabetes mellitus accounted for an increase of 19% and 37% in plasma NfL levels (adjusted model 2, **Table 2**). Contrastingly, fasting insulin and HOMA-IR were not significant predictors of plasma NfL levels in this cohort.

**Table 2:** Linear regression models for predictors of plasma neurofilament light (NfL) levels.

Unadjusted models		β-coefficient	95%CI		Adjusted R^2^	Model-p-value
			Lower	Upper		
Outcome variable	Predictors					
	log(Age)	1.208	0.807	1.609	0.2538	< 0.001
	Sex	-.1417	-2.996	0.016	0.0200	0.078
	Log(HOMA-IR)	0.039	-0.078	0.157	0.0005	0.506
Log(NfL)	Log(Insulin)	-0.029	-0.190	0.132	0.0013	0.722
	Log (glucose)	0.433	0.148	0.719	0.0710	0.003
	Diabetes status	0.180	0.081	0.285	0.1082	0.005
	Pre-diabetes	0.173	-0.009	0.356		0.063
	Type 2 diabetes	0.366	0.161	0.571		0.001
**Adjusted model 1**						
					0.2408	< 0.001
Log(NfL)	Diabetes_status					
	Pre-diabetes	0.070	-0.133	0.274		0.496
	Type 2 diabetes	0.271	0.019	0.523		0.035
	Log(age)	1.050	0.577	1.522		< 0.001
	Sex (females)	-0.015	-0.173	0.141		0.841
	Log(BMI)	-0.226	-0.758	0.305		0.400
	Log(HOMA-IR)	-0.072	-0.231	0.087		0.371
	Physical activity	-0.007	-0.00001	-0.00003		0.587
**Adjusted model 2**						
	Diabetes status				0.1641	0.001
	Prediabetes	0.198	0.021	0.375		
	Type 2 diabetes	0.377	0.180	0.573		
	Log(CRP)	0.123	0.045	0.201		0.002

Model 1 adjusted for age, sex, body mass index (BMI), homeostatic model assessment for insulin resistance (HOMA-IR) and physical activity; Model 2 adjusted for C-reactive protein.

## Discussion

This study reports for the first time that plasma NfL levels are associated with the clinical classification of diabetes status and glycemic control. The novel findings include (i) elevated levels of NfL in individuals with T2D and PD, (ii) diabetes diagnostic status as a significant determinant of plasma NfL levels even after adjusting for confounders such as age, sex, BMI, HOMA-IR, physical activity and CRP, (iii) plasma NfL levels positively correlated with fasting plasma glucose, (iv) no correlation between plasma NfL levels and fasting insulin and IR, suggesting that in people at risk of T2D and newly diagnosed T2D, glycaemic control may be the primary modifiable risk factor for neurodegeneration. Plasma NfL levels may be used to determine the risk of neurodegeneration in individuals with T2D and PD. Consistent with previously published reports, we found a significant positive association between age and plasma NfL levels (29–31). However, we did not find any association between plasma NfL levels with fasting plasma insulin or IR status in this cohort. Similarly, BMI and physical activity levels of the participants did not influence plasma NfL levels. In line with previous studies (28, 32), there was no association or accountability of sex on the plasma NfL levels between the diabetes diagnostic groups.

T2D has been associated with an increased risk of neurodegenerative diseases (33–35). Hyperglycaemia in T2D and PD is linked to neurodegeneration and structural brain abnormalities (lacunar infarcts, white matter hyperintensities, brain atrophy etc.) (7, 34, 36). Neurodegeneration is also evident in diabetic microvascular complications (37, 38). Several potential mechanisms between hyperglycaemia and neurodegeneration were previously published (9). These mechanisms include direct effects through advanced glycation end products, oxidative stress, IR and inflammation (9). Evidence suggests that peripheral nerve injury occurs during the early stages of the disease with mild glycaemic dysregulation (3). A prospective clinical study that included patients with pre-diabetes, and age- and sex-matched controls, indicated higher NfL mRNA levels in prediabetics than in controls (24). NfL mRNA levels were significantly higher in PD individuals with peripheral neuropathy than those without (24), suggesting that blood NfL mRNA levels could serve as a surrogate marker for early prediction of pre-diabetic peripheral neuropathy. Results from the current study substantiate the relationship between elevated plasma NfL levels with worsening glycaemic status (PD and T2D). Together, these observations indicate NfL levels could reflect the extent of neurodegeneration in those at risk and with overt T2D. Another cross-sectional analysis also showed elevated levels of plasma NfL in patients with inherited peripheral neuropathy compared to healthy controls (39). Plasma NfL levels were also linked to the severity of the disease (39). Although NfL may not be specific, it may be used to predict the severity of neurodegeneration across the spectrum of T2D disease progression.

Axonal damage is often evident in sub-clinical cerebrovascular diseases such as stroke (40), and this is identified by white matter lesions on magnetic resonance imaging (40). These observations have been shown to be predictive of clinical stroke (41). However, a blood test could be more economical and easier to detect these changes. A recent study found that serum NfL levels strongly predict incident stroke in study participants with diabetes who were stroke-free at baseline in a dose-dependent manner (42). The predictive capacity of NfL was substantially greater than established risk factors, with the highest quintile of NfL having an 11.4-fold higher hazard of stroke than the lowest quintile (unadjusted stroke hazards of other quartiles were 4.16, 4.91, and 6.49) (42). After adjusting for the Framingham stroke risk score and ethnicity, the highest quintile had a hazard ratio nearly 10 times greater than those in the lowest quintile (42). This study also reported that the addition of plasma NfL levels to the Framingham model significantly improved the discriminatory power of the Framingham risk score for stroke. Thus, indicating the importance of measuring serum NfL in T2D and those at risk of T2D for the prediction and possible early detection of diseases associated with neurodegeneration. In a cross-sectional analysis from the Swiss Atrial Fibrillation (Swiss-AF) cohort study (43), history of diabetes mellitus accounted for 26.8% increase in the serum NfL levels, similar to a magnitude of 10 years increase in age. While the mechanism remains unknown, a potential mechanism might involve diabetes induced neuronal damage.

Similar to other cross-sectional studies (44, 45), we have also replicated the observations of a positive association between age and plasma NfL levels. A strong association between age and NfL levels have also been demonstrated by a previous longitudinal study (32). Despite the positive correlation between NfL and age, glycaemic status remained a significant predictor of plasma NfL levels in the current study. However, individuals diagnosed with T2D indicated relatively higher plasma NfL levels than those with PD and normal glucose tolerance. The plasma NfL levels were reflective of the cut-offs presented in the younger population for neurodegenerative disorders. Diabetes is a progressive disease, and the risk increases with age, particularly in middle-aged adults (40-60 years, with an average onset of diagnosis at 45 years) (46, 47). Given the significant association of diabetes and neurodegeneration, age specific NfL levels will be required to determine the severity of neurodegeneration in people at risk and with T2D.

Insulin plays a crucial role in brain health, and IR can contribute to neurodegenerative disorders, such as AD and vascular cognitive impairment (48). Several studies have investigated the relationship between IR in the periphery and brain in AD and related disorders (49, 50). Moreover, in a non-diabetic population of AD subjects, HOMA-IR was associated with reduced cerebral glucose metabolism measured by [18F]FDG PET and with reduced grey matter volume (r^2^ = 0.37, p = 0.01) corrected for age and sex, suggesting IR has a significant impact on neurodegeneration (51). Another study evaluating CSF markers of AD, indicated no difference in CSF AD markers between individuals with IR and without IR, including CSF NfL levels (52). To date, there are no studies reported on the association between IR and plasma NfL levels. In the current study, we observed no association between plasma NfL levels and HOMA-IR. Further studies are required to validate these findings and determine the relationship between HOMA-IR and NfL.

Strengths of the current study include the first report on the association of plasma NfL levels with worsened glycaemic status, even after adjusting for age and other confounders. Furthermore, we also reported that plasma NfL levels is only associated with fasting plasma glucose levels, but not fasting plasma insulin levels. Including an arm with normal glycaemic control, PD and T2D, allowed us to study neurodegeneration (as measured by NfL) in individuals with varying glycaemic controls. This study also used a highly sensitive immunoassay for measuring plasma NfL levels. There are several limitations to the current study also such as the cross-sectional study design, with the inability to determine direct causality. Secondly, this study is a secondary analysis and thus lacks the exploration of other markers of neurodegeneration. Thirdly, the study cohort consists of trial subjects recruited as part of three different clinical trials carried out during the same period (2016-2017), and therefore the current findings may need validation in a single cohort with individuals of various glycaemic status. Fourthly, results from this study are preliminary and need further confirmation in larger and longitudinal cohorts to determine the utility of NfL in individuals with risk of T2D and overt T2D. Lastly, our study cohort also has an uneven number of individuals across the three groups. Recent findings indicate that blood NfL level might be partially affected by renal function (53). Renal function should be considered while assessing the relationship of NfL with other metabolic parameters. Therefore, future studies are warranted to examine the association between NfL and the risk or severity of neurodegeneration in T2D and its associated complications.

## Conclusion

Plasma NfL level is associated with diabetes status and plasma glucose levels but not plasma insulin levels or HOMA-IR in a cohort of middle-aged adults with PD and T2D. The addition of plasma NfL levels to the panel of existing clinical tests might improve the determination of risk of neurodegeneration in both people with risk of T2D and overt T2D. While these study findings are preliminary, if validated in longitudinal cohorts with a larger sample size, we believe plasma NfL levels could become a marker of severity of neurodegeneration in relation to worsening glycaemic status in diabetes mellitus.

## Data Availability Statement

The raw data supporting the conclusions of this article will be made available by the authors, without undue reservation.

## Ethics Statement

The studies involving human participants were approved by the Human Research Ethics Committee of the University of Newcastle, Callaghan, Australia, and written informed consent was obtained from all participants. The patients/participants provided their written informed consent to participate in this study.

## Author Contributions

RT, PC, and RM conceptualised the study. MG and RM provided resources and supervision of the project. SP measured plasma NfL. RT and JF recruited and collected data. RT and PC carried out the statistical analysis. RT interpreted the data and wrote the first draft. SP, PC, EH, JF, MG, and RM critically reviewed and edited the manuscript. All authors contributed to the article and approved the submitted version.

## Conflict of Interest

The authors declare that the research was conducted in the absence of any commercial or financial relationships that could be construed as a potential conflict of interest.

## Publisher’s Note

All claims expressed in this article are solely those of the authors and do not necessarily represent those of their affiliated organizations, or those of the publisher, the editors and the reviewers. Any product that may be evaluated in this article, or claim that may be made by its manufacturer, is not guaranteed or endorsed by the publisher.
